# NPM: latent batch effects correction of omics data by nearest-pair matching

**DOI:** 10.1093/bioinformatics/btaf084

**Published:** 2025-02-25

**Authors:** Antonino Zito, Axel Martinelli, Mauro Masiero, Murodzhon Akhmedov, Ivo Kwee

**Affiliations:** BigOmics Analytics, Via Serafino Balestra 12, Lugano 6900, Switzerland; BigOmics Analytics, Via Serafino Balestra 12, Lugano 6900, Switzerland; BigOmics Analytics, Via Serafino Balestra 12, Lugano 6900, Switzerland; BigOmics Analytics, Via Serafino Balestra 12, Lugano 6900, Switzerland; BigOmics Analytics, Via Serafino Balestra 12, Lugano 6900, Switzerland

## Abstract

**Motivation:**

Batch effects (BEs) are a predominant source of noise in omics data and often mask real biological signals. BEs remain common in existing datasets. Current methods for BE correction mostly rely on specific assumptions or complex models, and may not detect and adjust BEs adequately, impacting downstream analysis and discovery power. To address these challenges we developed NPM, a nearest-neighbor matching-based method that adjusts BEs and may outperform other methods in a wide range of datasets.

**Results:**

We assessed distinct metrics and graphical readouts, and compared our method to commonly used BE correction methods. NPM demonstrates the ability in correcting for BEs, while preserving biological differences. It may outperform other methods based on multiple metrics. Altogether, NPM proves to be a valuable BE correction approach to maximize discovery in biomedical research, with applicability in clinical research where latent BEs are often dominant.

**Availability and implementation:**

NPM is freely available on GitHub (https://github.com/bigomics/NPM) and on Omics Playground (https://bigomics.ch/omics-playground). Computer codes for analyses are available at (https://github.com/bigomics/NPM). The datasets underlying this article are the following: GSE120099, GSE82177, GSE162760, GSE171343, GSE153380, GSE163214, GSE182440, GSE163857, GSE117970, GSE173078, and GSE10846. All these datasets are publicly available and can be freely accessed on the Gene Expression Omnibus repository.

## 1 Introduction

Modern biomedical research uses high-throughput assays to generate single- and multi-omics data. For instance, RNA-sequencing data provides expression profiles of thousands of genes at genome-wide scale. Various experimental protocols at increasing granularity, including single-cell genomics, proteomics, or spatial transcriptomics have been developed and are now available. Yet, bulk RNA-seq continues to be a widely used assay in current research practices.

However, these advancements are accompanied by significant challenges. One such challenge is the high cost of sample collection, processing, and data generation, especially in studies involving a large number of samples (e.g. population-scale studies of disease). In large-scale studies, it is common practice to distribute the several steps of the data acquisition workflow across multiple centers. This often leads to the utilization of diverse protocols and technologies between centers. Additionally, research is increasingly relying on published datasets. Free, publicly available repositories like the Gene Expression Omnibus (GEO) database (Barrett *et al.* 2005), serve as valuable resources to scientists, offering quick access to existing datasets for re-analysis and to complement newly generated data.

Measurements in datasets generated in multiple centers will inevitably be affected by sources of technical variation, collectively known as “Batch Effects” (BEs). BEs may also arise within a single laboratory, due to distinct sequencing runs, depths, use of different sample donors, or when processing occurs in separate days. Cumulative variation can be also caused by smaller, hidden technical factors inherent to experimental settings. Altogether, BEs form a predominant, unwanted source of variation in omics data. BEs impact data mean and variance, and may confound real, underlying biological signal, altering false positive and false negative rates in downstream analyses, e.g. (Johnson *et al.* 2007, Leek *et al.* 2010, Kupfer *et al.* 2012, Tung *et al.* 2017, Čuklina *et al.* 2021, Phua *et al.* 2022). For instance, differential gene expression (DGE) testing may be affected by BEs. This is especially true in cases where the variable of interest is highly unbalanced between distinct batches. To minimize BEs, it’s crucial for the study design to involve a balanced representation of samples across batches. Unfortunately, study designs are often imperfect. When the variable of interest is highly imbalanced between batches, it is very challenging to disentangle biological signals from BEs.

Previous studies have assessed the extent to which BEs may impact measurements and discovery power, e.g. (Leek *et al.* 2010, Lauss *et al.* 2013, Leigh *et al.* 2018, Rasnic *et al.* 2019). Particularly in large datasets, BEs may underlie inconsistencies across studies. To address BEs computationally, batch correction methods have been developed. On a general level, these can be categorized into (i) “Supervised methods” such as ComBat (Johnson *et al.* 2007) and Limma RemoveBatchEffects (Ritchie *et al.* 2015) which uses a linear model to adjust known BEs; (ii) “Unsupervised methods,” such as RUV (Gagnon-Bartsch and Speed 2012), and SVA (Leek and Storey 2007) which attempts to identify potential sources of variation due to BEs without requiring prior knowledge of the batch vector. These methods mostly rely on specific assumptions or models. Distribution of biological data may often exhibit uncertain distortion from the model-expected distribution. Furthermore, batch correction methods suffer from the inherent heterogeneity both within and between batches. This is exacerbated in an unbalanced mix between study groups in the absence of matching replicates between batches. As a result, BE correction methods may not necessarily detect or adjust BEs adequately and consistently across diverse datasets. In order to achieve unbiased BE correction, both batch and phenotype labels would be needed. While this may be the case for fully controlled experiments, it’s unrealistic in clinical research where BEs may be unknown and phenotype classes undefined.

**Figure 1.**
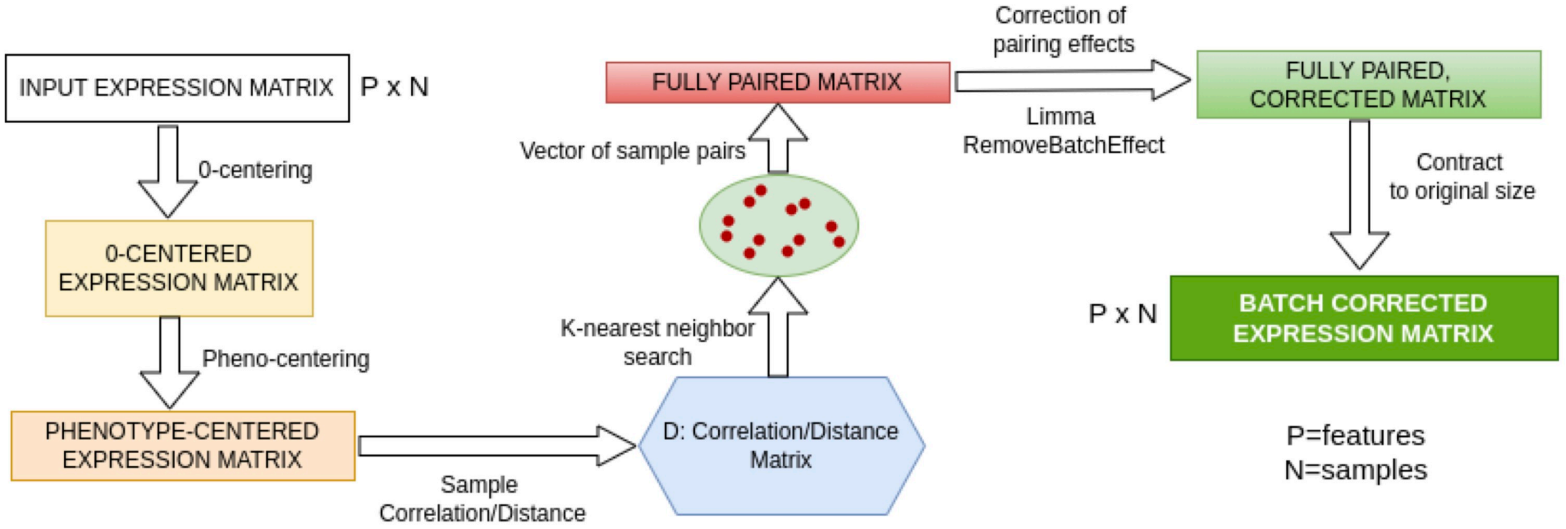
NPM algorithm workflow.

Here, we developed NPM (nearest-pair matching), a batch correction method that relies on distance-based matching to deterministically search for nearest neighbors with opposite labels, so-called “nearest-pair,” among samples (Fig. 1; Section 2). Our method was inspired by principles of the statistical matching theory (D’Orazio *et al.* 2006). Distinct matching methods have been made available through integrated frameworks. For instance, “MatchIt” (Ho *et al.* 2007, 2011) performs matching as a form of subset selection with pruning and weighting. NPM requires knowledge of the phenotypes but not of the batch assignment. NPM does not rely on specific models or underlying distribution. It does not require special experimental designs, randomized controlled experiments, control genes or batch information. NPM is based on the simple rationale that samples should empirically pair based on distance in biological profiles, such as transcriptomics. Our method generates a batch-corrected data matrix that can be used in downstream analyses.

We tested NPM in 11 publicly available transcriptomics datasets. We assessed multiple BE correction metrics, including number of differentially expressed genes (DEGs) between conditions of interest, principal component analysis, silhouette score for clustering, and nonlinear dimensional reductions. We demonstrate that NPM tackles BEs satisfactorily while preserving the biological heterogeneity between samples. NPM may outperforms commonly used batch correction methods, including Limma (Ritchie *et al.* 2015), ComBat (Johnson *et al.* 2007), SVA (Leek and Storey 2007), RUV (Gagnon-Bartsch and Speed 2012), and PCA (Jolliffe and Cadima 2016, Giuliani 2017).

## 2 Materials and methods

### 2.1 NPM algorithm

The input to NPM is a normalized and log-transformed gene expression matrix *X^p^*  ^×^  ^*n*^ (*p* = features, *n* = samples), which may suffer from noticeable or latent BEs. NPM does not require knowledge on the batches. Instead, NPM requires the phenotype vector. For a more efficient computation (beneficial for large datasets or when testing numerous datasets), NPM can select the top variable features (genes). By default, the 2000 most variable features are selected. The user can change this number based on specific needs. The top most variable set of features is used until we conduct distance calculation for nearest neighbor search (NNS). The fully paired dataset is then created and the full matrix returned (see below). The selected features are 0-centered and then further centered per condition group. Given *X^p^*  ^×^  ^*n*^, where n samples are distributed across *c* condition/phenotypic groups of biological interest, the rationale is to buffer potentially significant differences in average expression between the two phenotype groups (driven by or affecting the top genes) to improve unbiased estimation of correlation between samples. Inter-sample similarities are then determined by either computing the Pearson correlation matrix *D^n^*  ^×^  ^*n*^ (default) or Euclidean distance. *D* is transformed into a 1-D scale, handling both positive and anti-correlations. The Pearson correlation matrix *D^n^*  ^×^  ^*n*^ is subsequently decomposed into the c phenotypic/condition groups. For each sample, a *k*-nearest neighbor like search is conducted to identify the closest k-nearest samples across each *c* phenotypic/condition group. The *k*-NNS results into a matrix *X^n^*  ^× (^^*k*^  ^×^  ^*c*^^)^ where for each sample, *k*-nearest samples are identified per each c condition. The *X^n^*  ^× (^^*k*^  ^×^  ^*c*^^)^ matrix is then used to derive (i) a vector of length *L* = *n* × *k* × *c*, storing all the computed pairs; (ii) a fully paired dataset *X^p^*  ^×^  ^*L*^. The aim is to identify the closest samples within the opposite class to maximize the class effect while minimizing BEs. While is unlikely to have significant BEs in each pair, BEs may significantly emerge when analyzing all pairs into the same space. There could be systematic differences between pairs whose samples belong to distinct batches. These “pairing-effects” can be interpreted as systematic, batch-related differences emerging when comparing samples across different phenotypic groups in a pairwise manner. These effects would capture batch-related variations without explicit batch information. We then use Limma “RemoveBatchEffect” to correct for the “pairing effects” (Ritchie *et al.* 2015). The batch-corrected *X1^p^*  ^×^  ^*L*^ matrix is finally condensed into its original *p* × *n* size by computing, per each feature, the average values across duplicated samples. Thus, the *X1^p^*  ^×^  ^*n*^ matrix represents the batch-corrected dataset which can be used for further downstream analyses. Figure 1 shows the workflow of the algorithm (Fig. 1).

### 2.2 Datasets

NPM was tested on publicly available human RNA-seq datasets (Sprang *et al.* 2022) and a microarray dataset, and compared to Limma (Ritchie *et al.* 2015), ComBat (Johnson *et al.* 2007), SVA (Leek and Storey 2007), RUV (Gagnon-Bartsch and Speed 2012), and PCA. All datasets had available expression data and batch information. A brief description of each dataset is provided below.

- GSE120099 (Lo Sardo *et al.* 2018): Induced pluripotent stem cells generated from individuals carrying the 9p21.3 risk locus for coronary artery disease, and from nonrisk individuals. Genome editing was used to delete the haplotype, vascular smooth muscle cells (VSMCs) were generated and RNA-seq performed. Dataset for testing included a total of 92 samples (48 KO, 44 WT) split across 3 batches.
- GSE82177 (Wijetunga *et al.* 2017): RNA-seq from liver biopsies of 27 samples (10 uninfected controls, 9 HCV-infected nontumor samples, 8 HCV-infected HCC tumor samples) split across 2 batches. Control samples and nontumor samples were combined into a single group prior to BE assessment.
- GSE162760 (Farias Amorim *et al.* 2021): RNA-seq from whole blood samples from Leishmania braziliensis-infected individuals and noninfected controls. Dataset for testing included a total of 64 samples (14 noninfected controls, 50 Leishmania infected samples) split across 6 batches.
- GSE171343 (Bowles *et al.* 2021): RNA-seq from induced pluripotent stem cell-derived cerebral organoids carrying MAPT V337M mutation and CRISPR-corrected isogenic controls. RNA-seq performed at distinct differentiation stages. Dataset for testing included a total of 240 samples (100 V337M, 140 V337V) split across 3 batches.
- GSE153380 (Alvarez-Benayas *et al.* 2021): RNA-seq was performed on 5 primary Plasma Cells (PC), 28 Multiple Mieloma (MM) PC, and 5 cell line samples. Samples “A26.19” (PC) and “A27.22” (PC) appeared to be merged with A26.18 (PC) and A27.21 (PC), respectively, at source. For testing we included a total of 26 samples (23 MM, 3 PC) split across 3 batches.
- GSE163214 (Procida *et al.* 2021): RNA-seq was performed on HeLa Kyoto cells following knockdown of *JAZF1* and control cell lines. The following two samples were removed due to issues in downloading the data from the source: “GSM4975193_siJAFZ1_Rep2_Batch1” and “GSM4975199_siJAFZ1_Rep5_Batch2.” Dataset for testing included a total of 8 samples (5 controls, 3 KD) split across 2 batches.
- GSE182440 (Lim *et al.* 2021): RNA-seq was performed on postmortem putamen samples of control subjects and subjects affected with alcohol use disorder (AUD). Dataset for testing included a total of 24 samples (12 control, 12 AUD) split across 2 batches.
- GSE163857 (Moser *et al.* 2021): RNA-seq was performed from (i) microglia cells sorted from human-APOE carrying mice; (ii) microglia cells differentiated from human induced pluripotent stem cells from healthy subjects genotyped for APOE, untreated and treated with the heavy metals Cadmium (Cd) or Zinc (Zn). For testing, we included the 24 human microglia samples (15 control, 4 Cd-treated, 5 Zn-treated) split across 2 batches.
- GSE117970 (Cassetta *et al.* 2019): RNA-seq of purified monocytes and tumor-associated macrophages from breast cancer biopsies, endometrial cancer biopsies, and normal tissues. For testing we included a total of 88 samples (50 normal, 38 cancer samples) split across 5 batches.
- GSE173078 (Kim *et al.* 2021): RNA-seq was performed from gingival tissue biopsies in states of periodontal health, gingivitis, and periodontitis disease. Dataset for testing included a total of 36 samples (12 healthy control, 12 gingivitis, 12 periodontitis) split across 2 batches.
- GSE10846 (Lenz *et al.* 2008): Array expression profiling was performed on clinical samples from diffuse large B-cell lymphoma (DLBCL) patients. Dataset for testing included a total of 350 samples (167 ABC, 183 GCB), clustering into two pharmacological regimens (CHOP, R-CHOP).

### 2.3 Datasets preprocessing

All datasets were processed consistently within the same pipeline. For each dataset, the raw (un-normalized) data were downloaded from GEO (post RNA-seq alignment) along with associated metadata and processed in R v4 on a x86_64, Linux-gnu machine with Ubuntu 24.04 LTS. If feature (gene) identifiers were not official gene symbols, the official gene symbol was retrieved and assigned. In rare cases of duplicated gene symbols, the average expression values across duplicated features were calculated per sample, and duplicated features were removed. Genes undetected across all samples were removed. Expression data were normalized (i) within samples using counts per millions (CPM) followed by log2 + 1 transformation, and (ii) across-samples using quantile normalization in limma (Ritchie *et al.* 2015). Normalized data were used as input to the distinct batch correction algorithms.

### 2.4 Methods and metrics for BEs detection and correction

The following methods were used to assess BEs in the uncorrected datasets and upon batch correction:

Silhouette width score (SS): SS measures how well samples of the same group cluster together. SS values are defined within the range [−1,+1], where lower values indicate poor matching and clustering, and higher values indicating a good match. Thus, BEs could be assessed with SS, with higher values expected upon batch correction. SS were computed using the R package “cluster” (Maechler *et al.* 2024). The formula used to calculate SS for an individual data point is the following [Equation (1)]:

(1) $$\text{SS}\left(i\right)=\frac{b\left(i\right)-a\left(i\right)}{\text{max}\left(a\left(i\right),b\left(i\right)\right)}$$

where

a(*i*): average distance between point *i* and all other points of the cluster to which *i* belongs.

b(*i*): average distance between point *i* and all other points in the nearest cluster to which *i* does not belong.

We then computed the average SS across all data points within each dataset.

Signal-to-noise ratio (SNR) of Log2FC: SNR is a well-standardized measure in high-dimensional data, particularly genomic data. SNR measures the ratio between a signal of interest and a background noise. As signal, we utilize the average Fold-Change (FC) (in the Log2 scale) calculated through DGE analyses (see below) between the phenotypes/conditions of interest. The noise is defined as the average features’ standard deviation across all samples. The formula used to calculate SNR in a given dataset is the following [Equation (2)]:

(2) $$\text{SNR}=\frac{\text{averagelog}2\text{FC}}{\text{averageSD}}$$

PC1 ratio: Singular value decomposition is applied to the data matrix. For each phenotype class, the absolute Pearson’s correlation between each singular value and the phenotype label is computed (across all samples). In a dataset, we define PC1 Ratio as the ratio between the correlation value of the first PC and the sum of the correlation values of all available PCs. The higher the PC1 Ratio the better the batch correction. The formula used to calculate PC1 ratio in a given dataset is the following [Equations (3) and (4)]:

(3) $$\rho =\left|\text{cor}\left({\text{SVD}}_{s},\;\text{pheno}\_\text{labels}\right)\right|$$

*ρ* is run for each singular value, across all samples. Thus, there will be a *ρ* value for each singular value.

(4) $$\text{PC}1\;\text{ratio}=\frac{\rho \left[1\right]}{\sum \left(\rho \right)}$$

DGE testing: Appropriate batch correction should improve the signal to detect biologically meaningful differences between phenotypes/conditions of interest. This holds true both compared to uncorrected data and data with inefficient batch adjustment. On the basis of this principle, differential expression testing was performed between phenotypes/conditions of interest in both uncorrected data and upon batch correction using limma. DEGs are defined if absolute Log2FC ≥ 0.5 and FDR ≤ 0.05. Number of DEGs was used as a comparative metric between BE correction methods.

We sought to compute a score for each batch correction method. To this end, we first computed the ratio between number of DEGs, SNR, and SS of the corrected data versus the uncorrected data. As the uncorrected dataset was used as reference, the score is always 1 for the uncorrected data. The geometric mean of the ratios was then calculated as an integrated score of overall performance of each method in each dataset. Specifically [Equations (5) and (6)]:

(5) w=(ln(n.DEGs)+ln(SS)+ln(SNR))/n

 

(6) $$\text{Score}=e^{w}$$

where the denominator *n* is the number of integrating metrics and ln is the natural logarithm.

The higher each of the metrics, the better the clustering. Therefore, the higher the Score, the higher the overall quality of clustering and batch correction achieved in a given dataset. To have a metric representative of the overall method’s performance across all tested datasets, we computed the mean rank of the score for each method across all tested datasets.

## 3 Results and discussions

BEs represent a major source of unwanted variation in high-dimensional data. BEs mask meaningful biological signals across conditions of interest and can impact discovery and reproducibility. Technical sources of variation can be highly heterogeneous across datasets. They may remain undetected, and propagate across analyses. There is no common solution to correct BEs.

**Figure 2.**
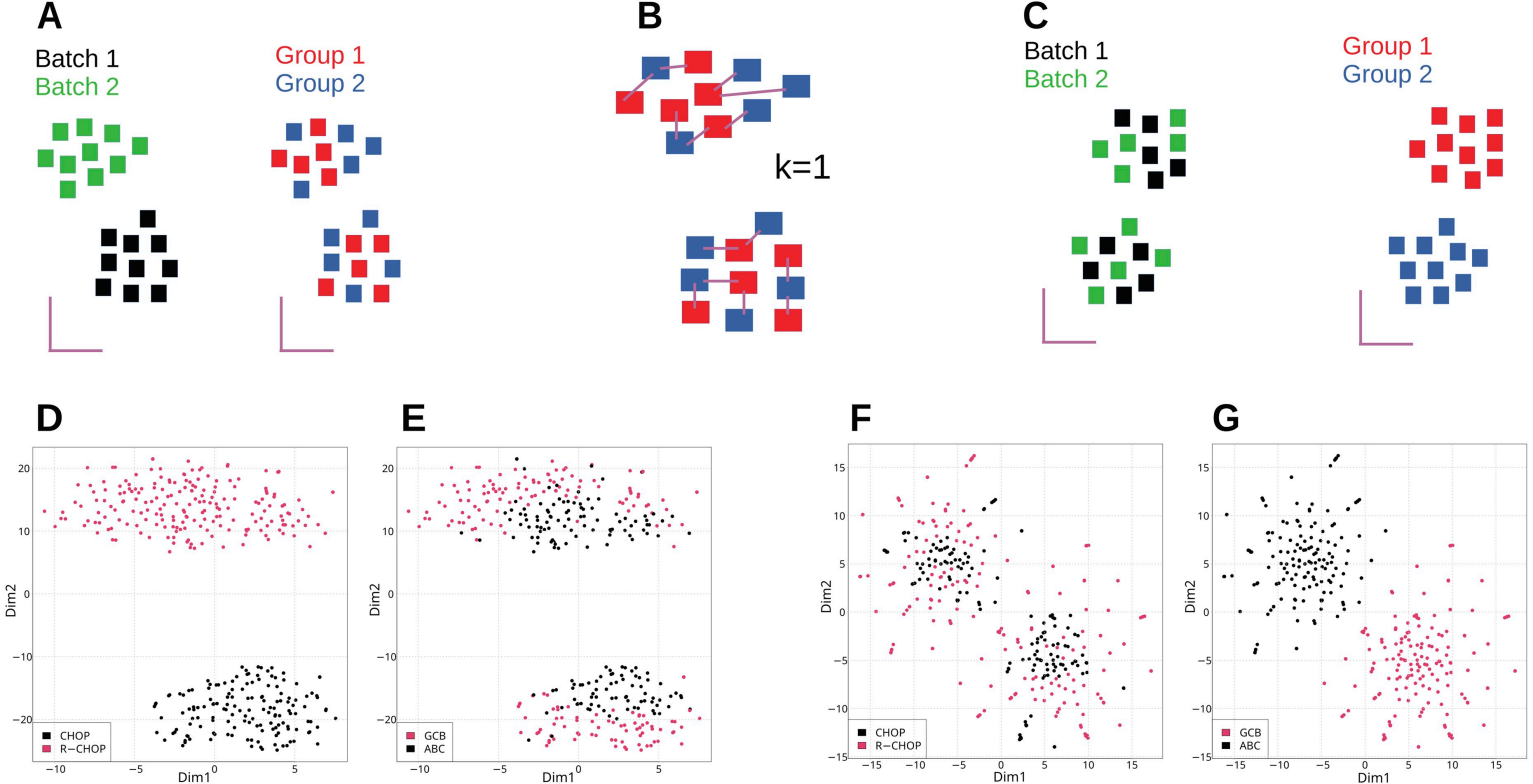
NPM algorithm and NPM testing. (A) Representative clustering of a dataset affected by BEs. (A) Samples segregate by batches rather than biological group. (B) NPM conducts NNS for each sample (Section 2). A *k* = 1 has been chosen as representative illustration. (C) NPM results into a batch-corrected dataset, where samples segregate by biological condition of interest. t-Distributed Stochastic Neighbor Embeddings (t-SNE) projections on the first two dimensions of uncorrected (D and E) and batch-corrected data (F and G) in a real dataset (GSE10846; Section 2).

In this work, we present NPM, a new method for BE correction (Figs 1 and 2A–C; Section 2). In line with principles of the statistical matching theory, NPM similarly performs unit (sample) selection to classify the units into the distinct phenotype groups. Subsequently, NPM conducts k-NNS through correlation or Euclidean distance between units. As NPM uses prior knowledge on phenotypic groups, it relies on a form of data stratification. Similarly, matching may also involve stratification, though with different modalities. The NNS results into pairs of units within and across condition classes. As NNS results into a fully weighted dataset (i.e. weight (distance) associated to each unit), the k closest units can be determined for each unit within each group. The NNS is nonparametric as it is neither based on propensity scores nor depends on regression parameters. Instead, it is based on sample distances within the stratified dataset, with pairs fully drawn from the original dataset. NPM enables full dataset matching: all available units are matched to k units in the group with opposite label. No units are dropped or removed.

NPM relies on the rationale that samples should empirically pair based on their distance in biological profiles. NPM is not restricted to prior assumptions on the nature of BEs, and it also works in studies where the requirement of balanced sample distribution among batches is violated. This, for instance, occurs in clinical research due to logistic and technical limitations. We tested NPM in 11 transcriptomics datasets spanning diverse scenarios in terms of sample size and balanced representation of samples between batches, and compared to supervised and unsupervised methods, including limma “RemoveBatchEffects,” ComBat, SVA, RUV, and PCA correction.

**Figure 3.**
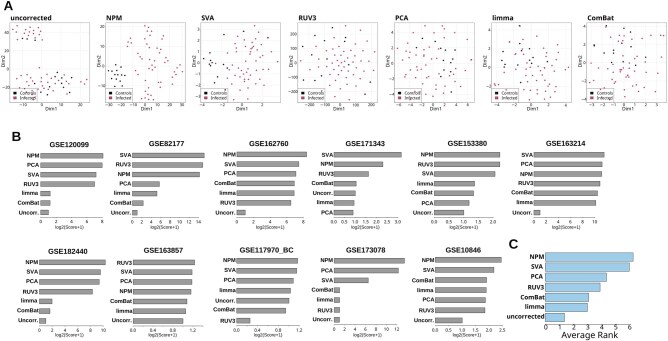
Comparison between NPM and other batch-correction methods. (A) t-SNE of uncorrected and batch-corrected data for GSE162760 (Section 2). In each plot the samples are colored by the phenotype variable. The batch correction method used is indicated at the top of each plot. (B) Bar plots of performance score (integrating multiple batch correction metrics; Section 2) for each batch correction method in each tested dataset. (C) Ranked bar plot of mean rank score (Section 2) for each BE correction method across all datasets.

We initially tested NPM on a large batched array expression dataset of activated B-cell (ABC) and germinal center B-cell (GCB) diffuse large B-cell lymphoma (DLBCL) samples (Lenz *et al.* 2008) clustered by pharmacological regimens (Fig. 2D and E). NPM successfully recovered the phenotype of interest, with samples clustering by DLBCL type, reflecting their biological heterogeneity (Fig. 2F and G). In another dataset (GSE162760; Section 2), NPM achieves improved batch correction while reasonably preserving the biological heterogeneity between samples compared to other methods (Fig. 3A). Upon batch-correction, samples part of the same phenotypic class cluster together.

To assess the extent to which batch correction impacts biologically meaningful signals in the data, we computed the number of DEGs between conditions of interest (#DEGs), SNR, silhouette score (SS), and correlation between principal components and phenotype labels, in the uncorrected data and following batch correction. Because we used average expression fold-change between phenotypes to compute SNR, greater cross-groups sample distances lead to higher SNR. Thus, SNR is positively correlated with resolution of phenotypes. SNR has been similarly used (Yu *et al.* 2023). However, SNR suffers of “lack of context,” as it does not inform about clustering quality or amount of batch-related noise removed. Differently, SS is a more direct proxy for clustering quality, as it measures the degree to which a sample is similar to its own cluster. A limitation of SS is that in presence of numerous clusters with varying size, it might not help interpreting inter-cluster differences. Computing SS can also be computationally expensive for large datasets. We also performed DGE testing between phenotypes and used the number of DEGs as an additional metric to assess batch correction. More DEGs should ideally be captured upon effective batch correction as phenotypes resolve correctly. However, caution should be applied as any significant difference in feature expression is context dependent, stemming from multiple independent phenotypes, but could also include false positives triggered by residual technical variation. Therefore, the above metrics can be used to assess batch correction with caution as each presents advantages and disadvantages. In order to have a single, more robust integrated score, we combined the distinct metrics. A higher integrated score would indicate a more effective overall batch correction within a dataset.

Assessment of t-SNE plots reveals that NPM is able to achieve improved clustering of samples based on the biological variable of interest, when compared to other methods (Supplementary Fig. S1). BEs appear substantially attenuated upon batch correction (Supplementary Fig. S2). As control, we also performed batch correction with all methods upon randomization of the phenotype classes. As expected, no appropriate batch correction was achieved (Supplementary Fig. S3). We found that NPM may outperform existing methods for most of the assessed metrics in the tested datasets (Supplementary Fig. S4). Likewise, NPM emerged among the top performing methods when combining the distinct metrics into a single, integrated score per each dataset (Fig. 3B; Supplementary Table S1; Section 2). We also computed a ranked score across all datasets (Section 2) and found that NPM exhibited overall improved performance (Fig. 3C; Supplementary Table S1). Therefore, when compared to other, highly used batch correction methods including Limma, ComBat, SVA, RUV, and PCA, NPM is capable to outperform or rank among the top methods. Altogether, the data indicate that NPM tackles BEs satisfactorily while also preserving the biological heterogeneity between samples. This is substantiated by (i) improved clustering of samples in the dimensionally reduced space and (ii) assessment of multiple, established batch correction metrics. NPM also preserves the original distribution of the data.

We applied NPM only to bulk transcriptomics data as the algorithm does not currently support single-cell level data. In fact, while NPM needs the phenotype labels (but not the batch labels), in single-cell RNA-seq data the phenotype labels—typically cell types—are initially unknown while the batch information is usually available. We believe NPM may also reasonably accommodate other high-dimensional, noisy data types, such as peptide and proteomics data. However, these data types are associated with other problems. For instance, it remains unclear whether preprocessing, including normalization and imputation, should be performed prior or after batch-correction in proteomics data. Thus, applying NPM to other data types warrants separate studies.

Importantly, whether using batch-corrected data or incorporating batch vectors in downstream analyses is another debated problem. The discussion has gained increased relevance with the advent of multi-omics. Generally, batch correction can be done in two alternative ways, i.e. by either generating a corrected data matrix that can be used in downstream analyses, or by incorporating batch vectors as covariates (e.g. in a linear model) to simultaneously perform batch correction and other analyses. Several batch correction methods, such as limma and ComBat, require a batch vector to generate a corrected data matrix. SVA, instead, requires the phenotype vector to infer surrogate variables that represent batch vectors for use in downstream analyses. Differently, NPM requires a phenotype vector for batch correction to generate a corrected data matrix; it does not output batch vectors. The advantage of removing BEs to generate a corrected data matrix is that the same, corrected data matrix can be used as input in all downstream analyses. However, whether batch correction is performed appropriately depends on the data type, its intrinsic features, and method used. Generally, risk of over-correction or introducing correlation structure in the data is significant. Differently, incorporating batch vectors into downstream analyses preserves the original data structure. However, how to perform adjustment for batch vectors could depend on the type of analyses; different analyses might need different correction methods which would lead to inconsistent results and lack of reproducibility. Addressing these problems is an interesting avenue for future research.

Given the inherent heterogeneity present in batched datasets, there may not be a single, all-encompassing solution to address BEs in biological data. Here, we propose NPM as a powerful alternative method, especially when other methods fail to resolve BEs.

## 4 Limitations

NPM relies on biological distances between samples. We show NPM may exhibit improved performance compared to highly used batch correction methods in a wide range of scenarios. Nevertheless, like any algorithm, it presents several limitations. First, it does not output batch vectors to be used in downstream analyses. Rather, it uses phenotype data to perform batch correction and to generate a batch-corrected data matrix for downstream analyses. As a general shortcoming of BE correction methods, there is always the risk of losing important biological heterogeneity. This may occur, for instance, when batch detection and correction fail to clearly distinguish batch-related variation from true biological variation when these are significantly intertwined or in presence of confoundings. NPM infers sample pairs based on intrinsic biological distances, however, mislabeling of phenotypes could impact this process. Loss of biological heterogeneity might also result from overcorrection. Second, NPM may not scale well for very large datasets, such as population-scale bulk RNA-seq data. This is due to the use of correlation and NNS sample by sample. Third, it does not handle datasets containing missing values. Internally, NPM uses limma RemoveBatchEffect to correct pairing effects. Unfortunately, limma RemoveBatchEffects (but also ComBat) is not designed to appropriately handle missing values. Therefore, NPM would require a complete matrix. For this reason, prior to NPM, data imputation is needed if missing values are present. As for all batch correction methods, it is a good practice to perform diagnostic checks on the data and conduct replication analyses to support results.

## Computing resources

Representative expression dataset of medium-large size: 20 174 features and 350 samples. On a Linux machine with 46G RAM, NPM tooks 8.4 secs, with total utilized RAM of 54 MiB and peak RAM of 858.4 MiB.

## Supplementary Material

btaf084_Supplementary_Data

## Data Availability

NPM is freely available on GitHub (https://github.com/bigomics/NPM) and on Omics Playground (https://bigomics.ch/omics-playground). Computer codes for analyses are available with no restrictions at (https://github.com/bigomics/NPM). The datasets used in this article are the following: GSE120099, GSE82177, GSE162760, GSE171343, GSE153380, GSE163214, GSE182440, GSE163857, GSE117970, GSE173078, GSE10846. All these datasets are publicly available and can be freely accessed on the Gene Expression Omnibus (GEO) repository.
