# Supplementary material for: NPM: latent batch effects correction of omics data by nearest-pair matching
Source: Bioinformatics. 2025 Feb 25;41(3):btaf084. doi: 10.1093/bioinformatics/btaf084 (PMC11925496; doi:10.1093/bioinformatics/btaf084)
Supplement: btaf084_Supplementary_Data [file btaf084_supplementary_data.zip › FigureS2.pdf]

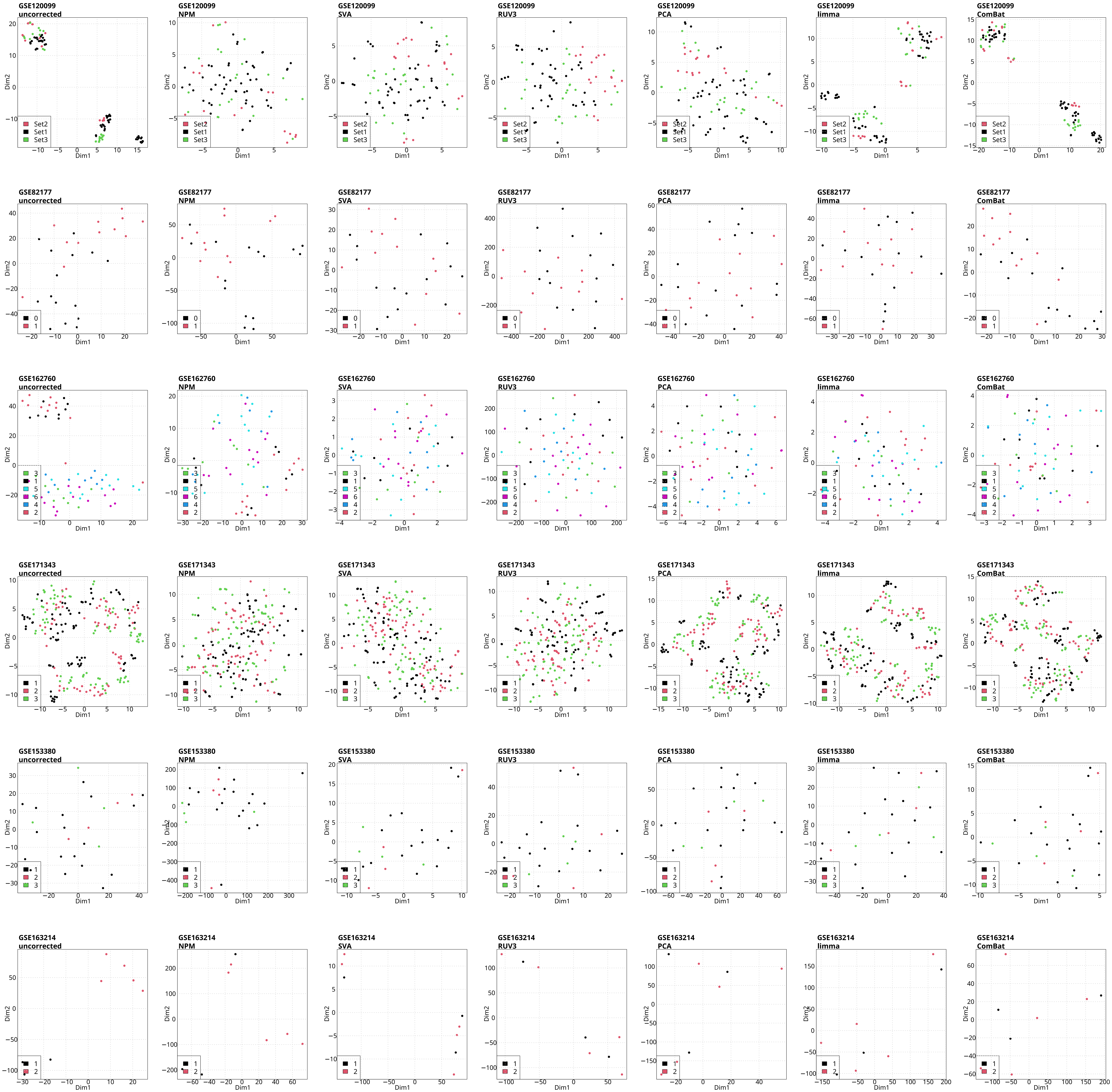
GSE120099 uncorrected
GSE120099 NPM
GSE120099 SVA
GSE120099 RUV3
GSE120099 PCA
GSE120099 limma
GSE120099 ComBat
Set2
Set1
Set3
GSE82177 uncorrected
GSE82177 NPM
GSE82177 SVA
GSE82177 RUV3
GSE82177 PCA
GSE82177 limma
GSE82177 ComBat
0
1
GSE162760 uncorrected
GSE162760 NPM
GSE162760 SVA
GSE162760 RUV3
GSE162760 PCA
GSE162760 limma
GSE162760 ComBat
3
1
5
6
4
2
GSE171343 uncorrected
GSE171343 NPM
GSE171343 SVA
GSE171343 RUV3
GSE171343 PCA
GSE171343 limma
GSE171343 ComBat
1
2
3
GSE153380 uncorrected
GSE153380 NPM
GSE153380 SVA
GSE153380 RUV3
GSE153380 PCA
GSE153380 limma
GSE153380 ComBat
GSE163214 uncorrected
GSE163214 NPM
GSE163214 SVA
GSE163214 RUV3
GSE163214 PCA
GSE163214 limma
GSE163214 ComBat
Dim1
Dim2

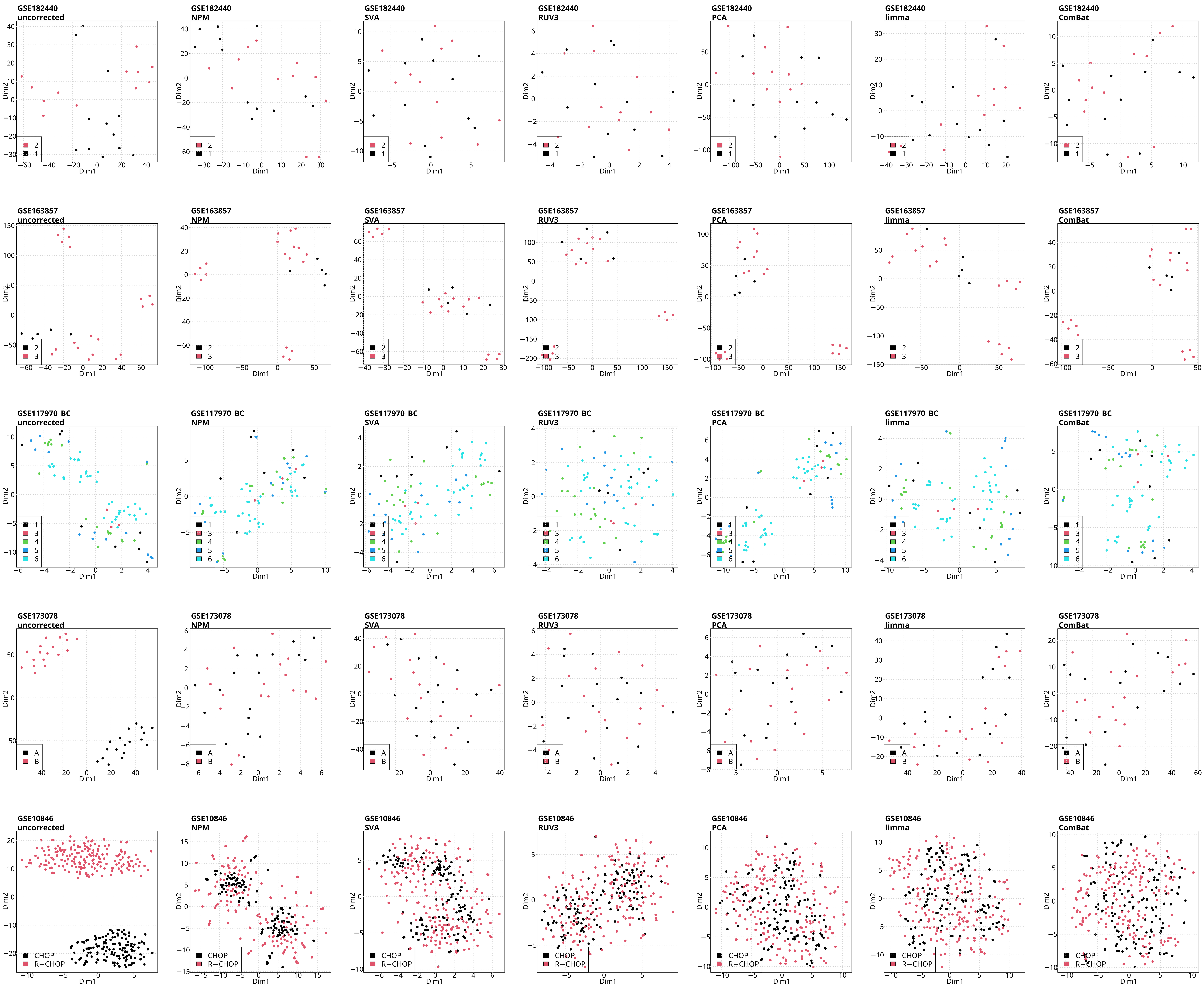

Figure S2. t-SNE plots of uncorrected and batch corrected data to assess clustering based on batch labels. The samples are colored by the batch labels as per each dataset's metadata. Dataset GEO identifier and batch correction method employed are reported at the top of each plot.
