# Supplementary material for: NPM: latent batch effects correction of omics data by nearest-pair matching
Source: Bioinformatics. 2025 Feb 25;41(3):btaf084. doi: 10.1093/bioinformatics/btaf084 (PMC11925496; doi:10.1093/bioinformatics/btaf084)
Supplement: btaf084_Supplementary_Data [file btaf084_supplementary_data.zip › FigureS1.pdf]

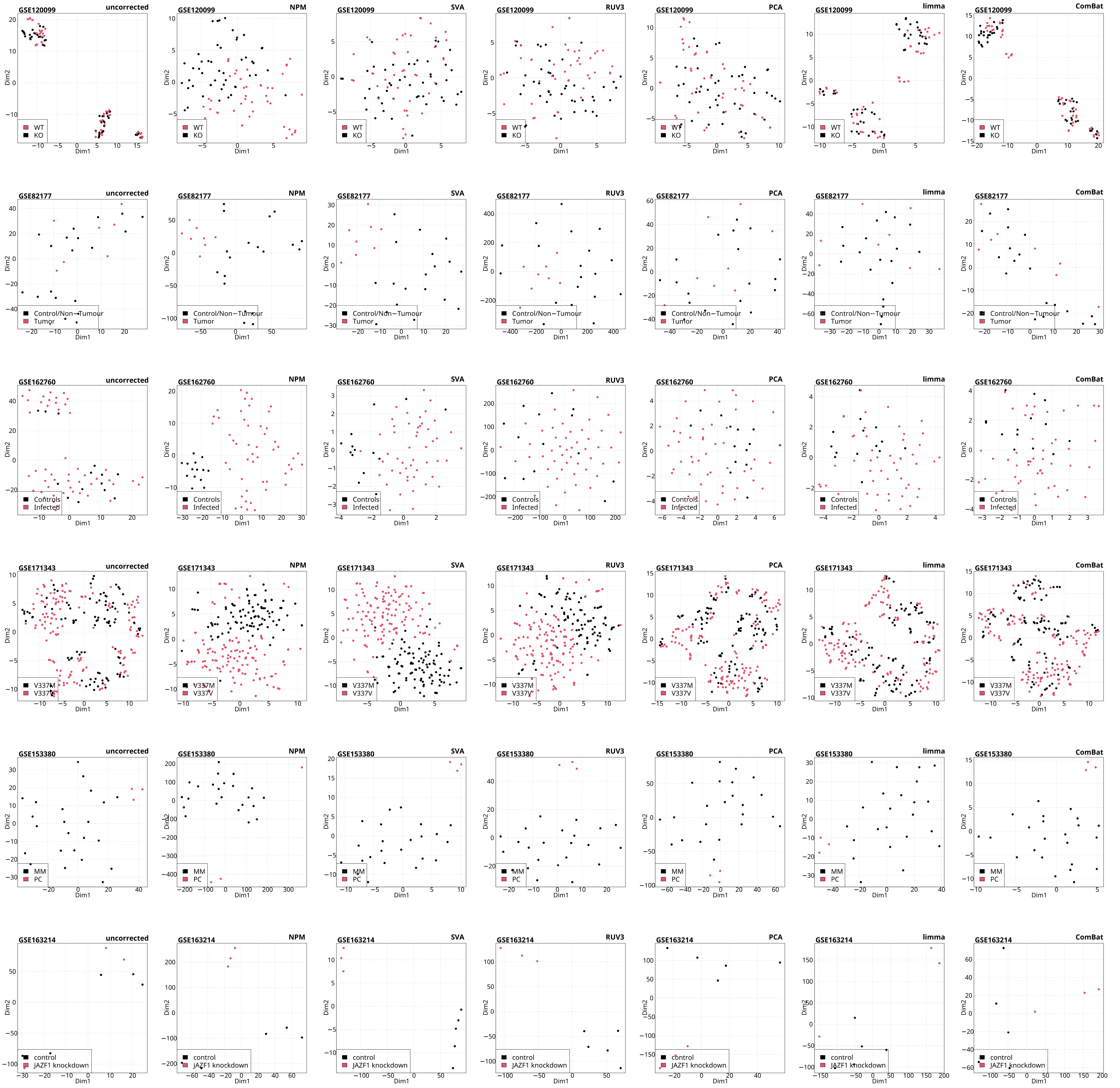

GSE120099
uncorrected
NPM
SVA
RUV3
PCA
limma
ComBat
WT
KO
GSE82177
Control/Non−Tumour
Tumor
GSE162760
Controls
Infected
GSE171343
V337M
V337V
GSE153380
MM
PC
GSE163214
control
JAZF1 knockdown
Dim1
Dim2

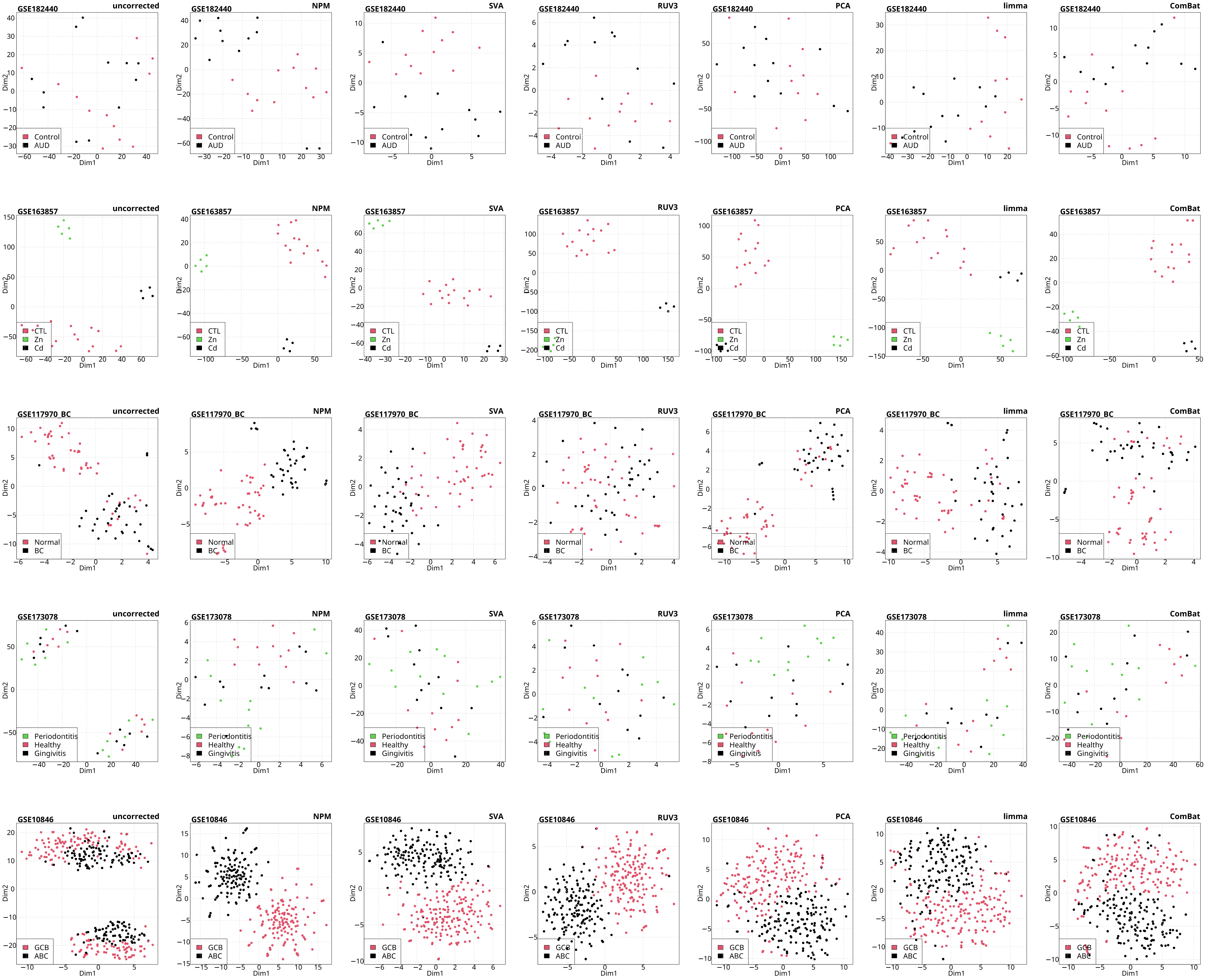

Figure S1. t-SNE plots of uncorrected and batch corrected data to assess clustering based on phenotype labels. The samples are colored by the biological variable of interest as per each dataset's metadata. Dataset GEO identifier and batch correction method employed are reported at the top of each plot.
