# Supplementary material for: NPM: latent batch effects correction of omics data by nearest-pair matching
Source: Bioinformatics. 2025 Feb 25;41(3):btaf084. doi: 10.1093/bioinformatics/btaf084 (PMC11925496; doi:10.1093/bioinformatics/btaf084)
Supplement: btaf084_Supplementary_Data [file btaf084_supplementary_data.zip › FigureS4.pdf]

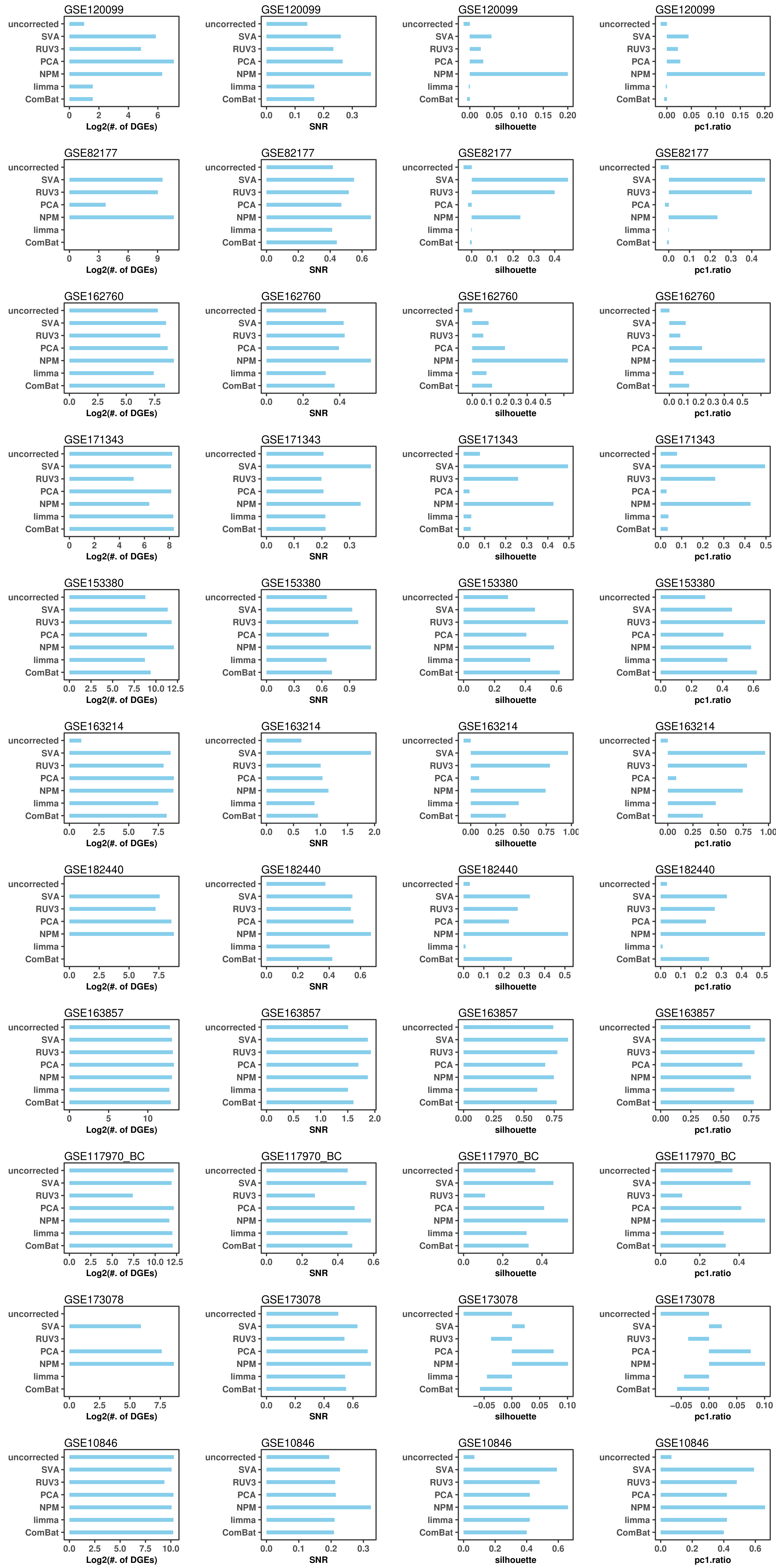

GSE120099
uncorrected SVA RUV3 PCA NPM limma ComBat
0 2 4 6
Log2(#. of DGEs)
0.0 0.1 0.2 0.3
SNR
0.00 0.05 0.10 0.15 0.20
silhouette
0.00 0.05 0.10 0.15 0.20
pc1.ratio
GSE82177
uncorrected SVA RUV3 PCA NPM limma ComBat
0 3 6 9
Log2(#. of DGEs)
0.0 0.2 0.4 0.6
SNR
0.0 0.1 0.2 0.3 0.4
silhouette
0.0 0.1 0.2 0.3 0.4
pc1.ratio
GSE162760
uncorrected SVA RUV3 PCA NPM limma ComBat
0.0 2.5 5.0 7.5
Log2(#. of DGEs)
0.0 0.2 0.4
SNR
0.0 0.1 0.2 0.3 0.4 0.5
silhouette
0.0 0.1 0.2 0.3 0.4 0.5
pc1.ratio
GSE171343
uncorrected SVA RUV3 PCA NPM limma ComBat
0 2 4 6 8
Log2(#. of DGEs)
0.0 0.1 0.2 0.3
SNR
0.0 0.1 0.2 0.3 0.4 0.5
silhouette
0.0 0.1 0.2 0.3 0.4 0.5
pc1.ratio
GSE153380
uncorrected SVA RUV3 PCA NPM limma ComBat
0.0 2.5 5.0 7.5 10.0 12.5
Log2(#. of DGEs)
0.0 0.3 0.6 0.9
SNR
0.0 0.2 0.4 0.6
silhouette
0.0 0.2 0.4 0.6
pc1.ratio
GSE163214
uncorrected SVA RUV3 PCA NPM limma ComBat
0.0 2.5 5.0 7.5
Log2(#. of DGEs)
0.0 0.5 1.0 1.5 2.0
SNR
0.00 0.25 0.50 0.75 1.00
silhouette
0.00 0.25 0.50 0.75 1.00
pc1.ratio
GSE182440
uncorrected SVA RUV3 PCA NPM limma ComBat
0.0 2.5 5.0 7.5
Log2(#. of DGEs)
0.0 0.2 0.4 0.6
SNR
0.0 0.1 0.2 0.3 0.4 0.5
silhouette
0.0 0.1 0.2 0.3 0.4 0.5
pc1.ratio
GSE163857
uncorrected SVA RUV3 PCA NPM limma ComBat
0 5 10
Log2(#. of DGEs)
0.0 0.5 1.0 1.5 2.0
SNR
0.00 0.25 0.50 0.75
silhouette
0.00 0.25 0.50 0.75
pc1.ratio
GSE117970_BC
uncorrected SVA RUV3 PCA NPM limma ComBat
0.0 2.5 5.0 7.5 10.0 12.5
Log2(#. of DGEs)
0.0 0.2 0.4 0.6
SNR
0.0 0.2 0.4
silhouette
0.0 0.2 0.4
pc1.ratio
GSE173078
uncorrected SVA RUV3 PCA NPM limma ComBat
0.0 2.5 5.0 7.5
Log2(#. of DGEs)
0.0 0.2 0.4 0.6
SNR
−0.05 0.00 0.05 0.10
silhouette
−0.05 0.00 0.05 0.10
pc1.ratio
GSE10846
uncorrected SVA RUV3 PCA NPM limma ComBat
0.0 2.5 5.0 7.5 10.0
Log2(#. of DGEs)
0.0 0.1 0.2 0.3
SNR
0.0 0.2 0.4 0.6
silhouette
0.0 0.2 0.4 0.6
pc1.ratio

Figure S4. Assessment of BE correction metrics for each method in each tested dataset. Bar plots show the number of differentially expressed genes (on the log2 scale) between the conditions of interest, signal-to-noise ratio, silhouette score, and correlation between principal components and phenotype labels (Methods), in the uncorrected data and following batch correction.
